# Mental rotation ability predicts the acquisition of basic endovascular skills

**DOI:** 10.1038/s41598-021-00587-x

**Published:** 2021-11-17

**Authors:** Katja I. Paul, Annegret Glathe, Niels A. Taatgen, Christopher J. Steele, Arno Villringer, Peter Lanzer, Fokie Cnossen

**Affiliations:** 1 Bernoulli Institute for Mathematics, Computer Science and Artificial Intelligence, University of Groningen, Nijenborgh 9, 9747 AG Groningen, The Netherlands; 2 Department of Neurology, Max-Planck Institute for Human Cognitive and Brain Sciences, Stephanstraße 1a, 04103 Leipzig, Germany; 3 Faculty of Medicine, University of Leipzig, Leipzig, Germany; 4 Department of Psychology, Concordia University, 7141 Sherbrooke Street West, Montreal, Quebec H4B 1R6 Canada; 5 Day Clinic for Cognitive Neurology, University of Leipzig Medical Center, Liebigstraße 16, 04103 Leipzig, Germany; 6 Berlin School of Mind and Brain, Humboldt-Universität zu Berlin, Unter den Linden 6, 10099 Berlin, Germany; 7 Center for Stroke Research Berlin, Charité Universitätsmedizin, Berlin, Germany; 8 Mitteldeutsches Herzzentrum, Health Care Center Bitterfeld-Wolfen GmbH, Friedrich-Ludwig-Jahn-Straße 2, 06749 Bitterfeld-Wolfen, Germany

**Keywords:** Human behaviour, Psychology, Cardiology, Interventional cardiology

## Abstract

Due to the increasing complexity of diseases in the aging population and rapid progress in catheter-based technology, the demands on operators’ skills in conducting endovascular interventions (EI) has increased dramatically, putting more emphasis on training. However, it is not well understood which factors influence learning and performance. In the present study, we examined the ability of EI naïve medical students to acquire basic catheter skills and the role of pre-existing cognitive ability and manual dexterity in predicting performance. Nineteen medical students practised an internal carotid artery angiography during a three-day training on an endovascular simulator. Prior to the training they completed a battery of tests. Skill acquisition was assessed using quantitative and clinical performance measures; the outcome measures from the test battery were used to predict the learning rate. The quantitative metrics indicated that participants’ performance improved significantly across the training, but the clinical evaluation revealed that participants did not significantly improve on the more complex part of the procedure. Mental rotation ability (MRA) predicted quantitative, but not clinical performance. We suggest that MRA tests in combination with simulator sessions could be used to assess the trainee’s early competence level and tailor the training to individual needs.

## Introduction

Since the introduction of endovascular interventions (EI) in the 1960s^1^ and 1970s^2^ they have become standard practice in cardiology and medicine^3^ as they are far less invasive and far more versatile than open surgery. Nevertheless, because the operator has to perform the procedure via a small incision, distant from the target site, with limited tactile feedback and suboptimal imaging guidance, the skills needed to master these procedures are complex and require extensive training and deliberate practice. More specifically, the operator is required to infer a 3D representation of the vascular system from the 2D fluoroscopy images. The orientation of the tools in the vascular system has to be derived from this representation that lacks depth cues^4^. Another factor that complicates EIs performance is the fact that they are conducted via tools such as catheters and guidewires as opposed to directly interacting with the arteries. Furthermore, EIs demand a high level of cognitive and decision-making skills, such as devising an interventional strategy, and selecting the tool types. This occurs dynamically and under uncertainty regarding the outcome of mechanical interactions between the tools and arterial walls. The need for instant decision making can also arise in unexpected events^5^.

Given the increasing cognitive and technical demands related to the expanding complexity of EIs due to technological advances and ongoing demographic changes, a deeper understanding of the required operators’ skills to achieve optimal outcomes is crucial. However, to date, neither the nature of operators’ skills required to perform EIs safely and efficiently nor the reasons for differences between the high and lower-level performers are understood well. To fulfil the aspirations of the interventional cardiovascular community to deliver excellent quality endovascular treatments, a number of important initiatives have been established world-wide by professional societies and associations, such as EuroPCR or the EAPCI^6,7^. The impact and efficacy of these important activities might be further enhanced by the development of interventional curricula based on the explication of experts’ skills and the design of protocols dedicated to deliberate practice of the required target skills^8^. To develop EI curricula based on deliberate skill acquisition, a better understanding of the factors that determine the performance quality is essential. In addition, knowledge of the extent to which EI performance depends on pre-existing ability, theorized here as primarily cognitive ability and manual dexterity, is expected to be important. If such abilities provide an advantage in acquiring endovascular skill, testing for them could benefit the individual training needs of candidates for endovascular medicine.

Earlier studies on minimally invasive procedures have found significant correlations between cognitive ability and manual dexterity tests and multiple simulator performance parameters^9–14^. As minimally invasive procedures are complex spatial tasks that require coordinated use of imaging and that are often associated with challenging spatial dilemmas, such as the fulcrum effect or inferring 3D structures from 2D images^13^, it is no surprise that tests of visual-spatial ability (VSA) predict performance (see^4^ for a systematic review). Often, a variety of tests are used to examine the relationship of different sub-concepts of VSA with performance in minimally invasive procedures, for example, visual working memory, spatial visualisation ability and mental rotation ability (MRA). However, in minimally invasive procedures MRA tests show the most robust relationship with performance, especially with faster skill acquisition^4^. Furthermore, as minimally invasive procedures also require coordinated fine motor control of both hands to manipulate the instruments, the link between manual dexterity and performance has been examined in multiple studies^9,15^.

A prospective study with 20 resident doctors examined the link between pre-existing psychomotor ability and the performance on simulated laparoscopy tasks^9^. Psycho-motor ability was measured with multiple VSA tests each measuring a different sub-concept, and multiple tests measuring different aspects of motor skill. The residents trained 1 h per week over the course of the academic year. The authors found that motor speed, manual dexterity, spatial scanning and visuo-spatial memory predicted the learning rate. Mental rotation ability predicted initial simulator performance. Based on the results, the authors concluded that dexterousness is crucial in laparoscopy, and that the mental rotation test used captured the VSA that is necessary to perform laparoscopy.

In a similar vein, a study was conducted to examine the relationship between baseline VSA, dexterity and veterinarian students’ performance on a simulated endovascular fluoroscopy task^16^. Here, VSA tests measuring MRA, pattern perception and spatial visualization were used as were multiple tests to measure dexterity. The simulation environment modelled a canine arterial system but was not validated. The authors found that only MRA predicted how quickly participants completed the endovascular fluoroscopy task. Manual dexterity did not, which is contrary with the previously discussed study^9^. As far as we are aware, only two studies have yet examined the link between cognitive ability and manual dexterity and endovascular skills^15,17^ on a validated simulator. The first study^15^ showed that participants who performed better on manual dexterity tests had shorter initial and final fluoroscopy times, administered less contrast-agent, and made fewer procedure-specific errors at the beginning of the endovascular training. Performance on a test that measured visual spatial memory predicted final basic and generic endovascular skills. The authors reasoned that the handier participants might have had more control over the endovascular tools and therefore manoeuvred them more efficiently through the vascular system. Superior visual spatial memory might have facilitated the visualisation of the vascular lesion. The second study^17^ showed that a fine motor and hand–eye coordination test, designed to assess the specific combination of these skills inherent to endovascular procedures, predicted performance in experienced clinicians but not in novice operators. In summary, MRA and manual dexterity might play a role in acquiring the necessary psychomotor skills to conduct an EI, however, based on the current literature, the exact relationship is not known.

Apart from VSA and fine motor skill, cognitive control is crucial to acquire the complex skills that are required to perform an EI^15^. One influential model of motor skill acquisition is the three-stage model postulated by Fitts and Posner^18^. The three stages are characterised as follows. During the cognitive phase, the learner gains insight into the different sub-components of the task. During the associative phase, these steps are carried out and the cognitive sequence of actions is paired with the actual psychomotor actions. Finally, in the autonomous phase the skill has become automatic. In terms of learning to perform an EI, the cognitive stage could comprise observing someone perform an EI, memorising the sequence of actions, and learning the purpose of each tool and the clinical guidelines. In the associative stage the procedure would be performed and gradually improved based on assessment and feedback^19^. Thus, during all learning phases, cognitive control and conscious attention are important as they allow for goal-directed actions and flexible adaptions. It follows, that both concepts may be especially important during deliberate practice to repeatedly perform an action, monitor one’s performance, actively learn from mistakes and to in-cooperate provided feedback^8^. Contrary to what might be expected, a recent review also stresses that cognitive control and conscious attention remain important, even after achieving an expert level^20^. In the studies conducted by van Herzeele et al.^15^ and Stefanidis et al.^9^, the relationship between these concepts and performance in minimally invasive surgery was examined using a compound test that measures visuo-spatial memory and cognitive control. They found that performance on the test correlated with shorter fluoroscopy times, faster skill acquisition and superior endovascular skills^9,15^. These results support the line of reasoning that cognitive control plays an important role in acquiring the skills needed to perform a minimally invasive procedure.

Based on the need of the interventional cardiovascular community to assure excellence in delivery of increasingly complex EI treatments, and building on the available research, we designed a study to determine the ability of medical students to acquire basic angiography skills following short-term simulator-based training and to explore whether manual dexterity, MRA, and cognitive control/conscious attention can predict the performance in a simulated internal carotid artery (ICA) angiography. We expected that participants would be able to acquire the basic angiography skills following short-term simulator training and that manual dexterity, MRA and cognitive control/conscious attention would be positively associated with simulator-based performance.

## Method

### Participants

Nineteen (9 female) medical students in the clinical years of medical studies at the Universities of Leipzig, Halle/Saale and Dresden, with a mean age of 23.9 ± 2.5 years participated in the study. The study was approved by the ethics committee of the medical faculty of the University of Leipzig (089/17-ek). All participants gave written informed consent according to the declaration of Helsinki. Participants had no prior experience with EIs and had not yet started the practical phase of medical training. Skilled musicians, athletes and gamers were excluded from the study as they might have an advantage in acquiring the psychomotor skills that are necessary to perform an angiography due to their visuo-motor training histories. Participants were reimbursed for their time at the end of the experiment.

### Cognitive ability and manual dexterity tests

To measure cognitive control and conscious attention we used the original Sustained Attention to Response Task (SART)^21^ downloaded from^22^. The instructions were translated to German. To measure manual dexterity we used the Grooved Pegboard^23^ and to measure mental rotation ability we used the Rot mental rotation test, described in more detail below. See Table 1 for an overview.

**Table 1:** Administered cognitive ability and manual dexterity tests.

Test	Test description	Skill measured	Outcome measure
Grooved Pegboard	The participant is asked to insert grooved pegs according to a specific order with one hand at a time into the 25 grooved holes of the pegboard. The grooved pegs fit into the grooved holes according to the key-keyhole principle. The participant is instructed to fill the pegboard as quickly as possible, while only picking up one peg at a time. The test comprises a right-hand and left-hand trial. In total, it took about 5 min to administer this test	Manual dexterity	Number of points calculated for the right- hand and left-hand trial separately as: the number of pegs inserted correctly plus the time it took in seconds plus the number of pegs that were dropped
Rot test	Computer-based task: Deciding whether two figures are identical, where one is shown from a different angle. This test took between 7 and 16 min, depending on how fast the participant responded	Mental rotation ability	Mean RT for correct trials and accuracy (% correct)
SART	Computer-based go-no-go task: Digits ranging from 1 to 9 are presented quickly (250 ms, followed by a 900 ms mask) at the centre of the screen. The task of the participant is to press the space bar every time a digit is shown apart from the digit 3. When the digit 3 is presented, the participant has to withhold his or her response. In total 225 digits were presented out of which 25 (11%) were the digit 3. Before the actual test started, a practice round was completed in which 18 stimuli were presented and feedback was given. During the actual SART, no feedback was provided. The actual SART took 4.3 min	Cognitive control/conscious attention	Mean RT for correct “Go” trials (where the participants had to press the space bar) and accuracy (% correct)

Description of the administered cognitive ability and manual dexterity tests and the skill that the particular test measured.

*RT* reaction time, *SART* sustained attention to response task.

### Rot test

In this mental rotation test, two artificial paperclip like figures are presented on screen, example stimuli are shown in Supplementary Fig. S1 (see Supplementary Materials 1). The stimuli were originally developed by Prof. Dr. Heinrich Bülthoff. The participant’s task is to decide whether these figures are identical. The difficulty is that the figures are depicted from different angles. In total, 46 stimuli were presented, the first 26 stimuli varied between difficulty levels one to three and the remaining 20 varied between difficulty levels four and five. Difficulty level 5 is the hardest. The difficulty level of the stimuli was defined by the degree of rotation of the stimuli. No more than three stimuli from the same difficulty level were presented consecutively and no more than three times after each other, both presented figures were identical (n = 22) or not identical (n = 24). Both targets were presented in a vertical column at the centre of the screen and participants had 15 s to indicate whether they were identical (right mouse button) or different (left mouse button). After the participant responded, a black screen appeared for 500 ms, followed by visual feedback presented for 2 s at the centre of the screen indicating whether the response was correct or incorrect.

### Material used for the simulator training

#### Endovascular simulator

**Figure 1:**
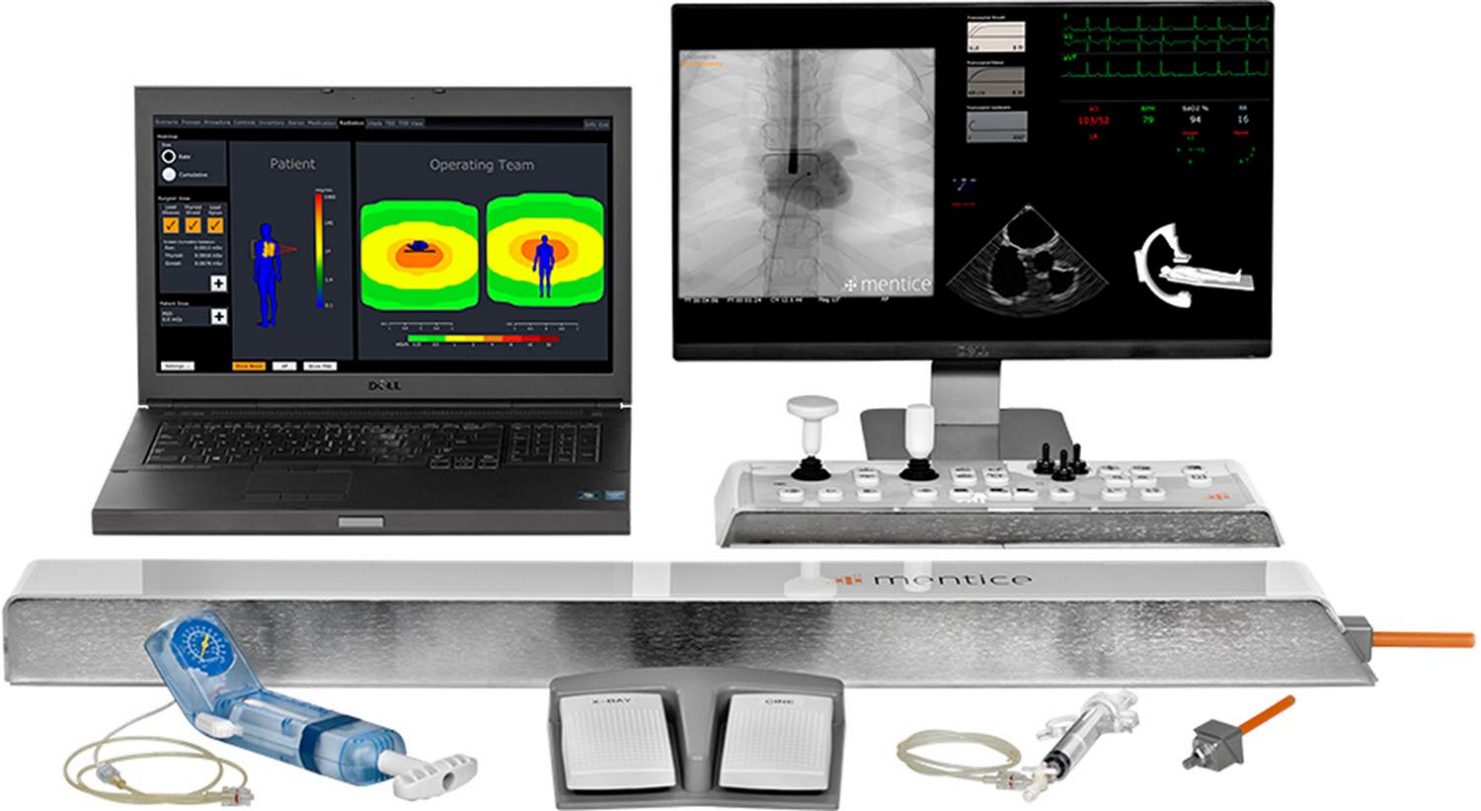
High fidelity endovascular simulator. The picture shows the endovascular simulator VIST G5 (Mentice Gothenburg, Sweden), which includes a laptop computer, a human body dummy, a control panel, a syringe for contrast-agent injection, a balloon indeflator and a food paddle to control the simulated X-ray.

The procedural skills training took place on the high-fidelity endovascular simulator VIST G5 (Mentice, Gothenburg, Sweden) described in^24^ and shown in Fig. 1. The screen capture device Live Gamer Portable 2^25^ was used to record participants’ performance by capturing the fluoroscopy screen. Prior to the training, participants read an instruction sheet that gave a brief background on endovascular procedures, mentioned the goal of the simulator training, described the steps of the procedure, the instruments to be used, clinical guidelines to take into account, and noted the performance metrics that would be measured. A general instruction video showed an expert (PL) performing the procedure on the simulator accompanied by audio commentary. There was a second instruction video to show how to use the catheter (Simmons 1) that would be used on the second and third training day.

### Procedure

**Figure 2:**
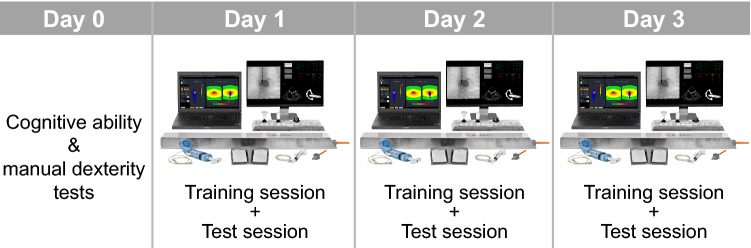
Experimental procedure. On day 0, two computer-based cognitive tests and the Grooved Pegboard were administered. On day one to three, participants individually completed training and test sessions on the simulator VIST G5 (Mentice, Gothenburg, Sweden).

The experimental procedure is shown in Fig. 2. Four days prior to simulator training, participants completed the three cognitive ability and manual dexterity tests (described in Table 1). To control for time-on-task effects, the order of the tests was counterbalanced across participants in such a way that each test was administered an equal number of times as the first, second and third one. Participants were tested individually and seated in front of the computer screen where all stimuli were presented. The cognitive ability and manual dexterity assessment session took approximately 45 min per participant.

#### Task performed on the simulator

The simulator training took place on three consecutive days. Participants performed a simulator-based aortic arch angiography using a pigtail catheter and a right internal carotid artery angiography using either a vertebral catheter (day 1) or a Simmons 1 catheter (day 2 and 3). To increase the complexity of the training across training days, participants trained on an aortic arch type I on day one, type II on day two and type III on day three over the course of the experiment. The type of an aortic arch is defined by the plane of the take-off of the supra-aortic arteries. The aortic arch type determines the choice of appropriate catheter^26^, the main difference between a vertebral catheter and a Simmons 1 is the shape of their tip.

#### Simulator training procedure

Training was performed in one-on-one individual sessions and given by KP, who was trained by the company that produces the endovascular simulator (Mentice, Gothenburg, Sweden) and an expert cardiologist (PL). On the first training day, participants read the written instructions and watched the general instruction video. Before training started, the experimenter asked participants to imagine that the simulator was a real patient and highlighted that (a) uncontrolled movements should be avoided and (b) that they should prioritise caution over speed. Each training day on the simulator comprised a training and a test session. The training session lasted for 60 min and feedback was given verbally, focusing first on major errors and then minor ones. After the training session, a test session of 20 min. followed, in which participants had to carry out the trained procedure on the same patient case that they practised on as well as and as often as possible without any feedback.

#### Performance evaluations

To obtain a detailed picture of participants’ EI performance we chose to use two complementary approaches: a *quantitative* one focusing on the number of errors made and the duration of a procedure, and a *clinical* one where performance was assessed by an expert cardiovascular interventionalist using clinical criteria.

Quantitative performance during the simulator training and test sessions was retrospectively evaluated by counting the committed errors on each trial using the screen captured sessions. Two trained raters first counted the committed errors independently, after which cases were discussed until consensus was reached. The raters were blind to participants’ performance on the cognitive and manual dexterity tests as well as to the expert’s clinical performance evaluation. The following basic error types were counted: movement of the catheter ahead of the guidewire; moving the patient table into the wrong direction; accessing the wrong blood vessel with the guide wire and/or catheter; tool not being in the field of view; and inadequate reference picture. The errors were added to yield the error score per trial (i.e. simulated intervention). The duration of a trial was automatically measured by the simulator. All trials on day 1–3 were rated. Failure to complete a single trial in the test session (this did not happen during the training) was penalized by assigning the maximum of the number of errors that was committed by other participants on the first trial; for duration the maximum of 20 min was used. Only in two out of 114 sessions on the simulator, did a participant fail to complete a single trial.

The expert cardiovascular interventionalist (PL) who was blinded to participants’ quantitative assessment and their performance on the cognitive and manual dexterity tests, performed the clinical evaluation of each of the test sessions for every participant. The expert split the procedure into two parts: the pigtail catheter placement in the aortic arch and the selective placement of the vertebral or Simmons 1 catheter into the ACC and ICA. Both parts of the procedure were rated separately on a five-point Likert scale (5 = perfectly acceptable, 1 = totally unacceptable). Failure to complete a single trial in the test session was penalized by assigning a rating of “1”. The Likert-scale rating focused on the guidewire and catheter handling, risky manoeuvres and whether the participant learned from previously committed mistakes.

### Design

The study was a prospective, randomized study with a longitudinal design where the quantitative and clinical performance measures across sessions was used to assess skill acquisition. The outcome measures from the cognitive ability and manual dexterity tests were used to predict the rate of improvement during the simulator sessions. The study was part of a larger study, comprising an additional control group and in which magnetic resonance imaging data were also acquired to examine brain plasticity related to endovascular skill acquisition. These data will be reported in another publication.

### Statistical analyses

Data were analysed using the statistical software R version 4.0.0 (2020-04-24)^27^. To create a combined score for the quantitative performance evaluation z-scores were calculated for the mean number of errors and mean duration of a procedure (i.e. trial) per participant and per simulator session. As both variables were skewed, they were log-transformed before calculating the z-scores. The sum of the z-scores for the number of errors and duration of a procedure was used as the quantitative performance outcome. The average ratings of each part of the procedure were used as the clinical performance outcomes. In order to combine reaction time and accuracy into one performance measure for the Rot test and the SART, the balanced integration score^28^ was calculated. The points for the left- and right-hand trial on the Grooved Pegboard were averaged to create a combined score for the Grooved Pegboard.

To examine whether the quantitative and clinical performance during the simulator sessions improved and whether any of the cognitive ability and manual dexterity tests predicted the rate of performance improvement, linear mixed effects models were built using the lme4 package^29^. The package lmerTest^30^ was used to test the significance of the parameters while the car^31^ package was used to test for multicollinearity of the predictors in a model. Furthermore, we used the EMAtools package^32^ to calculate Cohen’s d effect sizes. Three different models were created with quantitative performance as the dependent variable, *session* on the simulator (1, 2, 3, 4, 5, 6) as a fixed effect and performance on either the *Rot test*, the *SART,* or the *Grooved Pegboard* as a second fixed effect. A random intercept was included for participant. As each day on the simulator comprised a training and a test session, there were six sessions in total on the simulator, which were evaluated separately. Furthermore, six models were created with the clinical performance of the aortic arch angiography or the cannulation of the ACC/ACI as the dependent variable, a fixed effect of *test session* (1, 2, 3), and the performance on either the *Rot test*, the *SART* or the *Grooved Pegboard* as a second fixed effect. Again, a random intercept for participant was included. We also tested for interactions and random slopes to examine whether any of the cognitive ability or manual dexterity tests predicts the rate of improvement differently across simulator sessions and to allow the learning rate to differ across participants. We used a forward stepwise model fitting procedure and determined the better fitting model for the fixed effects based on p-values while we determined the random effect structure by comparing models using the Likelihood-ratio test; a more complex model was only chosen if it explained significantly more variance. Spearman rank correlations were used to compute correlations between the cognitive and manual dexterity tests and the quantitative and clinical performance evaluations as well as between the latter two variables. Statistical parameters with a p-value below 0.05 were regarded as statistically significant.

## Results

### Quantitative performance and prediction

**Figure 3:**
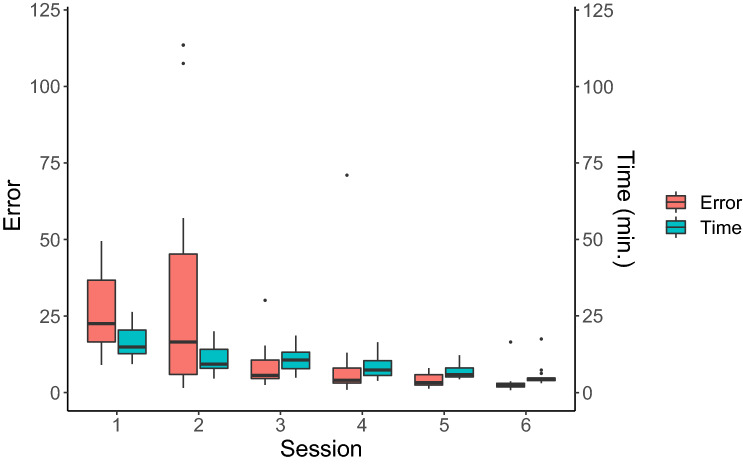
Quantitative performance. Boxplot representing the quantitative skills assessments: the median number of errors and median duration of a procedure per simulator session (session 1–6 refer to training session 1, test session 1, training session 2, test session 2, training session 3, test session 3, respectively) are indicated by the horizontal line in the box. Within the box, the middle 50% are shown, the whiskers depict the range from the 25th percentile minus 1.5 times the interquartile range (IQR) and 75th percentile plus 1.5 times the IQR. The circles show the outliers. This figure was created using the R software package^27^.

The linear-mixed effect models revealed that participants’ performance increased across sessions. The mean decrease in the quantitative performance from one session on the simulator to the next was significant (β = − 0.61, *p* = 2e^−16^, smaller scores indicate better performance; Fig. 3 shows the median duration and number of errors per training and test session. The variance inflation factor was < 4 in all three models indicating that there was no multicollinearity between any predictors in any model. Furthermore, the mean decrease in the quantitative performance on the simulator as the score on the Rot test increases by one (higher scores indicated better performance on the Rot test), was significant (β = − 0.26, *p* = 0.0032). This means that participants who performed better on the Rot test improved more rapidly during the training on the endovascular simulator. When correcting for conducting three tests (two cognitive and one manual dexterity tests that served as predictors), the p-value of the Rot test as a predictor is still below 0.05. Neither the performance on the SART nor on the Grooved Pegboard predicted participants’ rate of improvement and there were no significant interactions. Descriptive statistics of the quantitative evaluation are shown in Supplementary Table S1, while Supplementary Table S2 shows the descriptive statistics of the performance on the cognitive ability and manual dexterity tests. Supplementary Table S3 shows a correlation matrix of all outcome variables used in these three models, while Supplementary Table S4 shows the statistical models (Supplementary Table S1–S4 can be found in Supplementary Materials 2).

### Clinical performance evaluation and prediction

**Figure 4:**
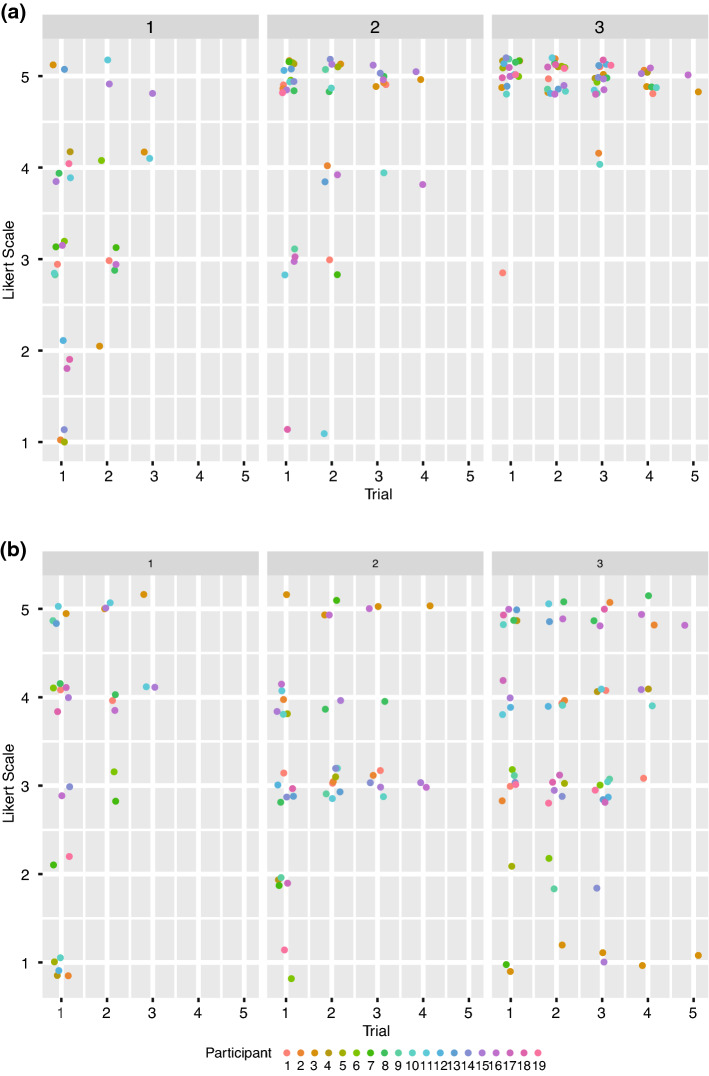
Clinical performance evaluation of the angiographies. (**a**) Shows the clinical rating of participants’ performance of the passage of the pigtail catheter into the aortic arch in test session 1–3. On day 3, 95% of all trials completed by all participants of the pigtail catheter placement were rated with maximal performance (“totally acceptable”). (**b**) Shows the clinical rating of participants’ performance of accessing the ACC/ICA in test session 1–3. On day 3, 17% of all trials of the catheter placement into the carotid artery were still rated with “unacceptable and totally unacceptable”. Likert scale rating 1 = totally unacceptable, 5 = perfectly acceptable. Both figures show all individual trials completed by all participant during the three test sessions. Data points were jittered for improved clarity. This figure was created using the R software package^27^.

The linear-mixed effect models of the clinical performance evaluation of placing the catheter into the aortic arch revealed that participants’ performance increased across the test sessions (see Fig. 4a). The mean increase in the Likert-scale rating from one test session to the next was significant (β = 0.98, *p* = 1.84e^−07^, higher ratings indicate better performance). Neither the performance on the Rot task, the SART nor on the Grooved Pegboard predicted participants’ learning rate in placing the catheter into the aortic arch and there were no significant interactions. The linear-mixed effect models of cannulation the ACC/ACI revealed that the clinical evaluation of participants’ performance did not increase across the test sessions (p = 0.51, see Fig. 4b). None of the cognitive and manual dexterity tests predicted participants’ rate of improvement in cannulating the ACC/ACI and there were no significant interactions. The variance inflation factor of all six models was < 4 indicating that there was no multicollinearity between any predictors in any model. Descriptive statistics of the clinical evaluation per test session are shown in Supplementary Table S1, while Supplementary Table S5 shows the correlation between the clinical evaluation of the different test sessions with the cognitive and manual dexterity tests as well as the quantitative evaluation. All statistical models are provided in Supplementary Table S4 (see Supplementary Materials 2).

## Discussion

The goal of the present study was to examine whether participants naïve to endovascular procedures can acquire basic catheterisation skills with limited simulator training. Further, we examined whether pre-existing cognitive abilities and manual dexterity play a role in acquiring these skills. In order to gain insight into participants’ training-related improvement we evaluated performance quantitatively (number of errors, duration of a procedure) and clinically (expert assessment). The quantitative assessments showed that EI naïve participants can acquire some basic catheterisation skills within three daily simulator training sessions. However, the picture becomes more complex when evaluating participants’ performance clinically, based on the execution of procedural steps and overall catheter handling. While there was consistent improvement in the passage of the pigtail catheter into the aortic arch and performing the aortic arch angiography, there were still large individual differences in performing the cannulation of the ACC/ACI followed by angiography in the last test session (see Fig. 4b, Supplementary Table S1). Participants’ individual performances remained inconsistent across the three tests sessions. This finding indicates that the quantitative assessment captures basic skills that suffice to perform the simple procedure of an aortic arch angiography successfully and consistently. However, these basic skills are not sufficient to perform the technically more complex angiography of the ACC/ACI consistently with success. The clinical performance assessment revealed for example that two participants adopted high-risk manoeuvres (rapid, uncontrolled push without guidewire guidance) when placing the catheter into the carotid artery. One participant persistently cannulated the subclavian along with the vertebral artery. These performance fluctuations seem to be due to an incomplete grasp of the basic mechanics of the catheter over the guidewire advancement technique, required for successful performance of these more complex procedural steps. Furthermore, the heterogeneity of the performance was likely also due to the increased difficulty of the catheter placement into the carotid artery due to the type III aortic arch on the last training day. Hence, the data suggest that with increasing technical difficulty, a deeper understanding of the principles of the EI becomes crucial. Consequently, more detailed explanations of the individual procedural steps, clarification of the sources of errors, and means for their corrections seem to be required. For example, once the ostium of the brachiocephalic trunk has been found, the critical importance of the distance between the tip of the guidewire and the catheter tip, the guidewire hold, and the speed of the catheter advancement need to be detailed, practiced and committed errors corrected by feedback. Furthermore, the quantitative and the expert evaluation both confirmed the large variability in participants’ performance during the simulator training (e.g., Supplementary Table S1, Figs. 3, 4). These differences appear to indicate that participants used different strategies when learning this complex task. Specifically addressing these differences during teaching might become crucial to a better understanding of the task and thus to training success.

The second goal of this research was to examine whether pre-existing cognitive abilities and manual dexterity play a role in acquiring endovascular skill. The ease and speed with which the MRA test was performed predicted the rate of change in the quantitative performance on the simulator. Good MRA might have allowed participants to more quickly grasp the orientation of the tools in the vascular system and thus, may have led to more efficient movements. These findings are in line with previous research that has linked MRA to performance on simulator-based minimally invasive procedures^9–14^. Furthermore, the results fit Ackerman’s^33^ findings concerning individual difference predictors in complex skill acquisition. More specifically, spatial abilities are predictive of the rate of skill acquisition during the cognitive stage of learning, the stage in which the learner is gaining insight into the task and learns how to approach it^18^. In contrast, MRA did not predict clinical performance. This suggests that MRA tests might be useful to identify individual training needs, while clinical evaluations uncover other aspects of learning, which may include being able to learn from mistakes or risk-taking behaviour. Looking closer into the differences between the quantitative and clinical assessment, it became apparent that the quantitative evaluation focused more on metrics concerning lower-level skills in guidewire and catheter handling, controlling the patient table and the quality of the image acquired. Clinical evaluation emphasised higher-level factors that may compromise the patients’ safety, such as committing high-risk manoeuvres (e.g. rapid thrust of the catheter into the target artery ahead of the guidewire). These aspects of learning could be further addressed using the principle of deliberate practice by discussing, explaining and correcting the errors of individual candidates during additional simulator sessions^8^.

Contrary to our expectations, manual dexterity assessed with the Grooved Pegboard predicted neither quantitative nor clinical performance on the simulator. Interestingly, previous work that investigated the relation of multiple VSA sub-categories and fine motor dexterity with fluoroscopic skills in veterinary students also found that only MRA predicted performance^16^. We hypothesise that this outcome is due to the fact that the main difficulty in our task (cannulating the ICA), was navigating the tools in a 3D environment under 2D guidance, exactly like in the task used in^16^. Hence, our evidence indicates that this part of an EI heavily relies on MRA, while manual dexterity is of lesser importance than, for example in treating a renal artery stenosis using an endovascular technique^15^. Furthermore, EIs require additional visual-motor skills that are not measured by the Grooved Pegboard, such as inferring depth despite the lack of depth cues and executing actions in a perpendicular plane. It would be interesting to examine the association of the performance on our training task with the psycho-motor skill test that was developed by^17^ specifically to assess visual-motor skills that are required to perform endovascular procedures.

Performance on the SART^21^ predicted the rate of improvement in neither the quantitative nor in the clinical performance evaluation. One reason for the lack of predictive relationship seems to be the fact that there was little variation in performance on the SART between participants. Homogenous performance among participants decreases the probability of finding a significant predictive relationship. Furthermore, participants were very motivated and enthusiastic to acquire this new medical skill and therefore might have actively focused on improving their task performance on the endovascular simulator. Thus, participants’ engagement in the task, along with a high-level of cognitive control per se among participants might have led to the lack of relationship between performance on the SART and performance on the endovascular simulator.

An important limitation of our study is the fact that only one expert performed the clinical evaluation of the test sessions. This means that, while consistent, the expert rating might be biased and should therefore be interpreted with caution. At the same time, it is interesting that the expert’s clinical rating gave unique insight into the more pragmatic clinical aspects of the task. Another limitation of our study is that the training was simulator-based, which implies that we cannot generalise our results directly to the clinic. Additional limitations are the relatively modest size of the cohort and the short training duration. We cannot rule out the possibility that relationships between the cognitive and manual dexterity tests and endovascular skill acquisition might be weaker or stronger with a larger sample size, or a longer training duration. Future research should investigate the association of simulator performance with MRA in a longer study, where residents follow a controlled training program over the course of multiple weeks. Such work should include a test battery, including additional tests that measure visual search, visual attention and visual-spatial memory e.g.^34^ and a test of psychomotor ability like the one described by^17^ prior to training. This would help to expand upon our findings and shed light on the question of whether MRA continues to predict performance in the long run. Furthermore, a replication study with a larger sample would also allow to test whether the statistical models used in the current study still fit or whether multiple different models are needed to explain participants’ learning strategies. A larger sample size would also make it possible to use e.g. clustering approaches, where participants could be grouped by their learning strategies. Such an analysis may provide more insight into individual differences in learning, the role of MRA in this context and might also provide a basis for personalized training regimes in future studies^35^.

In summary, our results show that EI naïve participants can acquire basic catheterization skill during a three-day simulator training. When evaluating performance, both quantitative and clinical assessment seem of critical importance to gain full insight into which aspects are difficult to learn and which are associated with pre-existing cognitive ability and manual dexterity. MRA tests, in combination with simulator training might be useful to identify the trainee’s early competence level and tailor the training to individual needs. Similarly, simulator training sessions could serve as a platform to detect differences in strategies employed by the trainees when learning to conduct EIs safely. This information could then be used to personalize training to correct maladaptive strategies or specifically support the respective strategies. Thus, explicit training of basic catheter skills, such as interpreting fluoroscopic images and navigating the tools efficiently could take place on a simulator, before moving on to the catheterisation-laboratory to warrant patient safety and create a safe environment for the learner.

## Supplementary Information

Supplementary Information.

## Data Availability

The datasets and the analysis of the current study are available from the corresponding author on reasonable request.

## Abbreviations

EI: Endovascular intervention
VSA: Visual-spatial ability
MRA: Mental rotation ability
SART: Sustained Attention to Response Task
ICA: Internal carotid artery
ACC: Common carotid artery
PL: Peter Lanzer (co-author)
