# Supplementary material for: Mental rotation ability predicts the acquisition of basic endovascular skills
Source: Sci Rep. 2021 Nov 17;11:22453. doi: 10.1038/s41598-021-00587-x (PMC8599481; doi:10.1038/s41598-021-00587-x)
Supplement: Supplementary file 1 — Supplementary Information. [file 41598_2021_587_MOESM1_ESM.docx]

**Supplementary Materials for**

**Mental rotation ability predicts the acquisition of basic endovascular skills**

**Katja I. Paul*^a, d^, Annegret Glathe^d, g^, Niels A. Taatgen^a^, Christopher J. Steele^d, e^ , Arno Villringer^c, d, f, h^, Peter Lanzer^b^ and Fokie Cnossen^a^**

*Bernoulli Institute for Mathematics, Computer Science and Artificial Intelligence, University of Groningen, The Netherlands^a^, Mitteldeutsches Herzzentrum, Health Care Center Bitterfeld-Wolfen GmbH, Bitterfeld-Wolfen, Germany^b^, Day Clinic for Cognitive Neurology, University of Leipzig Medical Center, Leipzig, Germany^c^, Department of Neurology, Max-Planck Institute for Human Cognitive and Brain Sciences, Leipzig, Germany^d^, Department of Psychology, Concordia University, Montreal, Canada^e^, Berlin School of Mind and Brain, Humboldt-Universität zu Berlin^f^, Faculty of Medicine, University of Leipzig, Leipzig, Germany^g^, Center for Stroke Research Berlin, Charité Universitätsmedizin, Berlin, Germany^h^*

Corresponding author: Katja Isabel Paul*

Email: [k.i.paul@rug.nl](mailto:k.i.paul@rug.nl)

**Supplementary Materials 1**

**Supplementary Figure S1.**

**Stimuli from the Rot mental rotation test**

a) Shows a stimulus with difficulty level 1, the shapes are different, b) Shows a stimulus with difficulty level 5 stimulus, the shapes are identical. These stimuli were originally developed by Prof. Dr. Heinrich Bülthoff.

**Supplementary Materials 2**

**Supplementary Table S1.**

Descriptive statistics of the quantitative and clinical performance evaluation of all sessions on the endovascular simulator.

|  | *Quantitative performance evaluation* | | | | *Clinical performance evaluation* | |
| --- | --- | --- | --- | --- | --- | --- |
|  | *Error* | | | *Time* | *Rating aortic arch angiography* | *Rating ICC/ICA angiography* |
|  | *M, S.D.* | | *M, S.D.* | | *M, S.D.* | *M, S.D.* |
| Training Session 1 | 26.7 ± 13.6 | 16.2 ± 5.14 | | | N.A. | N.A. |
| Test Session 1 | 33.4 ± 38.2 | 10.9 ± 4.76 | | | 2.98 ± 1.21 | 3.13 ± 1.53 |
| Training Session 2 | 8.36 ± 6.33 | 10.9 ± 3.70 | | | N.A. | N.A. |
| Test Session 2 | 8.69 ± 15.5 | 8.49 ± 4.22 | | | 4.27 ± 1.13 | 3.00 ± .99 |
| Training Session 3 | 4.09 ± 1.96 | 6.7 ± 2.15 | | | N.A. | N.A. |
| Test Session 3 | 3.02, ± 3.36 | 5.14 ± 3.16 | | | 4.95 ± .13 | 3.40 ± 1.15 |

*Note:* N.A. stands for not applicable as the expert only performed the clinical performance rating for the test sessions of each training day on the endovascular simulator. M indicates the mean and S.D. the standard deviation, n = 19 for each session.

**Supplementary Table S2***.*

Descriptive statistics of the reaction time and accuracy data from the cognitive tests and the number of points on the manual dexterity test.

|  | *Rot Test* | *SART* | *Grooved Pegboard* |
| --- | --- | --- | --- |
|  | *M, S.D.* | *M, S.D.* | *M, S.D.* |
| RT | 6,146.00 ± 1034.67 | 437.05 ± 75.02 | Right hand: 88.00 ± 7.01 |
| Accuracy | 69.00 % ±7.54% | 97.00 % ± 2.18% | Left hand: 92.42 ± 10.15 |

*Note:* RT stands for reaction time and is displayed in milliseconds, accuracy is the percentage correct, for the Grooved Pegboard the number of points for the left- and right-hand trial are shown instead of RT and accuracy. M stands for mean and S.D. for standard deviation.

**Supplementary Table S3.**

Correlation matrix showing the correlation between the cognitive ability and manual dexterity tests with the clinical performance evaluation per simulator session.

|  | Cognitive and manual dexterity tests | | |
| --- | --- | --- | --- |
|  | Rot test | SART | Grooved Pegboard |
| Training Session 1 | -.55 | -.19 | -.08 |
| Test Session 1 | -.42 | .02 | .07 |
| Training Session 2 | -.32 | -.22 | -.16 |
| Test Session 2 | -.51 | .05 | -.01 |
| Training Session 3 | -.64 | -.08 | .16 |
| Test Session 3 | -.15 | .12 | .05 |

*Note:* These values show the Spearman rank correlation between the performance on the six simulator sessions and the cognitive and manual dexterity tests.

**Supplementary Table S4**.

Coefficients of the linear-mixed effects models of the quantitative and qualitative performance on the simulator.

| Model 1: Quantitative Performance ~ Session + Rot test + (1\|subject) R^2^_marginal_ = .61, R^2^_conditional_ = .67 | | | | | |
| --- | --- | --- | --- | --- | --- |
| Parameter | Estimate | *t* | *p* | Cohen’s d | 95% CI |
| Intercept | 1.51 |  |  |  |  |
| Session (1-6) | -.61 | -13.64 | 2e-^16^* | -2.81 | (-.69, -.52) |
| Rot test (z-score) | -.26 | -3.43 | .00321* | -1.66 | (-.41, -.11) |
| Model 2: Quantitative Performance ~ Session + SART + (1\|subject) R^2^_marginal_ = .54, R^2^_conditional_ = .67 | | | | | |
| Intercept | 1.51 |  |  |  |  |
| Session (1-6) | -.61 | -13.64 | 2e^-16^* | -2.81 | (-.69, -.52) |
| SART task (z-score) | -.07 | -.44 | .67 | -.21 | (-.40, .26) |
| Model 3: Quantitative Performance ~ Session + Grooved Pegboard + (1\|subject) R^2^_marginal_ = .54, R^2^_conditional_ = .67 | | | | | |
| Intercept | 2.34 |  |  |  |  |
| Session (1-6) | -.61 | -13.64 | 2e^-16^* | -2.81 | (-.69, -.52) |
| G. Pegboard | -.01 | -.47 | .64 | -.23 | (-.05, .03) |
|  |  |  |  |  |  |
| Model 4: Qualitative Performance of the Aortic arch angiography ~ Test session + Rot test + (1\|subject) R^2^_marginal_ = .44, R^2^_conditional_ = .44 | | | | | |
| Parameter | Estimate | *t* | *p* | Cohen’s d | 95% CI |
| Intercept | 2.10 |  |  |  |  |
| Test session (1- 3) | .98 | 6.40 | 1.84e^-7^* | 2.10 | (.68, 1.28) |
| Rot test (z-score) | .14 | 1.61 | .125 | .78 | (-.03, .31) |

| Model 5: Qualitative Performance of the Aortic arch angiography ~ Test session + SART + (1\|subject)  R^2^_marginal_ = .41, R^2^_conditional_ = .44 | | | | | |
| --- | --- | --- | --- | --- | --- |
| \| Parameter \| Estimate \| *t* \| *p* \| Cohen’s d \| 95% CI \| \| --- \| --- \| --- \| --- \| --- \| --- \| | | | | | |
| Intercept | 2.10 |  |  |  |  |
| Test session (1-3) | .98 | 6.40 | 1.84e^-7^* | 2.10 | (.68, 1.28) |
| SART task (z-score) | -.07 | -.47 | 0.64 | -0.23 | (-.39, .23) |
| Model 6: Qualitative Performance of the Aortic arch angiography ~ Test session + Grooved Pegboard test + (1\|subject) R^2^_marginal_ = .41, R^2^_conditional_ = .44 | | | | | |
| Intercept | 2.34 |  |  |  |  |
| Test session (1-3) | .98 | 6.40 | 1.84e^-07^* | 2.10 | (.68, 1.29) |
| G. Pegboard | .00 | -.144 | 0.89 | -.07 | (-.04, .03) |
| Model 7: Qualitative Performance of the ACC/ICA angiography ~ Test session + Rot test + (1\|subject)  R^2^_marginal_ = .03, R^2^_conditional_ = .06 | | | | | |
| Parameter | Estimate | *t* | *P* | Cohen’s d | 95% CI |
| Intercept | 2.91 |  |  |  |  |
| Test session (1- 3) | .13 | .67 | .51 | .21 | (-.26, .52) |
| Rot test (z-score) | .12 | 1.09 | .29 | .53 | (-.09, .35) |
| Model 8: Qualitative Performance of the ACC/ICA angiography ~ Test session + SART + (1\|subject)  R^2^_marginal_ = .06, R^2^_conditional_ = .06 | | | | | |
| Intercept | 2.91 |  |  |  |  |
| Test session (1-3) | .13 | .67 | .51 | .18 | (-.25, .52) |
| SART task (z-score) | .32 | 1.69 | .09 | .46 | (-.05, .69) |
| Model 9: Qualitative Performance of the ACC/ICA angiography ~ Test session + Grooved Pegboard + (1\|subject) R^2^_marginal_ = .01, R^2^_conditional_ = .06 | | | | | |
| Intercept | 3.80 |  |  |  |  |
| Test session (1-3) | .13 | .67 | .51 | .22 | (-.26, .52) |
| G. Pegboard | -.01 | -.42 | .68 | -.20 | (-.06, .04) |

*Note:* The estimates represent the change in quantitative or qualitative performance on the simulator when the parameter increases by 1. Abbreviations: ACC/ICA: common carotid artery/ internal carotid artery, SART; sustained attention to response task. G. Pegboard stands for Grooved Pegboard. R^2^marginal indicates the R^2^value based on the fixed effects, while R^2^conditional includes the fixed and random effects. * *p* < .005.

**Supplementary Table S5.**

Correlation matrix showing the correlation between the cognitive ability and manual dexterity tests with the clinical performance evaluation of the aortic arch -and ACC/ICA angiography and the correlation between the quantitative and clinical performance evaluation.

|  | Cognitive and manual dexterity tests | | |  |
| --- | --- | --- | --- | --- |
|  | Rot test | SART | Grooved Pegboard | Quantitative evaluation |
| Test Session 1 Aortic arch | .38 | .09 | .02 | -.59 |
| Test Session 1 ACC/ICA | .18 | .46 | .00 | -.80 |
| Test Session 2 Aortic arch | .06 | -.38 | .40 | -.28 |
| Test Session 2 ACC/ICA | .18 | .10 | .08 | -.74 |
| Test Session 3 Aortic arch | -.32 | -.11 | .39 | .17 |
| Test Session 3 ACC/ICA | .22 | .07 | -.28 | -.23 |

*Note*: The values display the Spearman rank correlation between the variables. High values on the clinical evaluation and low values for the quantitative evaluation indicated good performance. Thus, a negative correlation indicates accordance between both performance evaluations.
